# Co‐Rumination as a Moderator Between Best‐Friend Support and Adolescent Psychological Distress

**DOI:** 10.1002/jad.12483

**Published:** 2025-02-16

**Authors:** Steffie van der Mey‐ Baijens, Patricia Vuijk, Kim Bul, Pol A. C. van Lier, Marit Sijbrandij, Athanasios Maras, Marieke Buil

**Affiliations:** ^1^ Research Centre Innovations in Care Rotterdam University of Applied Sciences Rotterdam the Netherlands; ^2^ Department of Clinical Neuro and Developmental Psychology Vrije Universiteit Amsterdam Amsterdam the Netherlands; ^3^ Amsterdam Public Health Research Institute ‐ Mental Health Amsterdam UMC Amsterdam the Netherlands; ^4^ Research Institute for Health and Wellbeing Centre for Intelligent Healthcare Coventry University Coventry United Kingdom; ^5^ ARQ National Psychotrauma Centre Diemen the Netherlands

**Keywords:** adolescence, anxiety symptoms, co‐rumination, depressive symptoms, dyadic support, perceived stress symptoms

## Abstract

**Introduction:**

Co‐rumination, characterized by excessively discussing problems and dwelling on negative affect within a dyadic friendship, has been associated with adolescents' symptoms of depression, anxiety and perceived stress‐collectively referred to as psychological distress. This study explored whether co‐rumination moderates the association between perceived best friend support and psychological distress.

**Methods:**

The study included 187 adolescents (52.9% girls; 88.0% Dutch ethnic background) recruited from two cohorts between March 2017 and July 2019. Assessments took place at two time points: symptoms of depression, anxiety, and perceived stress were assessed via self‐report measures at the final grade of primary school (T1; *M*age = 11.8 years) and in secondary school (T2; *M*age = 13.3 years). Co‐rumination and perceived best friend support were measured via self‐report in secondary school.

**Results:**

Findings indicate that best friend support was associated with lower psychological distress and conversely, co‐rumination was associated with higher psychological distress while adjusting for prior distress symptoms. Moderation analysis revealed that moderate levels of co‐rumination (relative to the samples mean) decreased the positive effects of perceived best friend support on symptoms of depression (**B** = 0.06, SE = 0.03, 95% CI [0.00, 0.11], *p* = 0.05, β = 0.11) and perceived stress (*B* = 0.06, SE = 0.01, 95% CI [0.03, 0.08], *p* = 0.000, β = 0.10). At very high levels of co‐rumination (relative to the samples mean), best friend support exacerbates perceived stress.

**Discussion:**

This study underscores the potential negative impact of co‐rumination in supportive peer relationships and recommends promoting awareness of the risk of co‐rumination while building a repertoire of (dyadic)emotion regulation strategies.

The transition from late childhood into early adolescence is marked by rapid physical changes, cognitive and emotional maturation, as well as changing and expanding social contexts (Brown and Larson 2009; Yurgelun‐Todd 2007). This challenging period contributes to an increase in symptoms of depression, anxiety, and stress, collectively referred to as psychological distress (Boer et al. 2022; Twenge et al. 2019). Concurrently, adolescents develop greater autonomy from parents and increasingly seek support from peers (Brown and Larson 2009; Furman and Buhrmester 1992), highlighting the importance of peers as an important outlet for discussing feelings and problems and as a source for social support in challenging situations (Bokhorst et al. 2010; Furman and Buhrmester 1992). Both theoretical and empirical studies suggest that feeling supported by friends and classmates enhances self‐esteem, self‐confidence and self‐efficacy, and are associated with higher levels of well‐being and less psychological distress during adolescence (Chu et al. 2010; Cohen and Wills 1985; Graber et al. 2016; Schwartz‐Mette et al. 2020).

However, some studies that specifically examined support between early adolescent best friends, have not found an association with psychological distress (Ling et al. 2021; Lyell et al. 2020; van der Mey‐Baijens et al. 2022). These paradoxical results might be explained by the function and form of the support that is provided. That is, some adolescents might support each other by engaging in “co‐rumination,” by some described as a dyadic emotion regulation strategy to gain insight in ones problems and alleviate negative affect (English and Eldesouky 2020; Hazel et al. 2023; Stone et al. 2022). Co‐rumination is characterized by extensively discussing, rehashing, and speculating about ones problems as well as dwelling on negative affect (Rose et al. 2017). Indeed, several studies show that co‐rumination is associated with elevated internalizing problems during adolescence (Bastin et al. 2021; Harrington 2020; Spendelow et al. 2017).

Thus, friendships in adolescence characterized by higher levels of perceived support and self‐disclosure are often assumed to protect adolescents against the development of psychological distress (Costello et al. 2024; Schwartz‐Mette et al. 2020). However, studies have also shown that when adolescents discuss their feelings, emotions, or problems through co‐rumination, the effects of perceived friend support may become less beneficial and instead might even be associated with elevated levels of distress (Rose et al. 2014; Tilton‐Weaver and Rose 2023).

## 1 Co‐Rumination, Perceived Best Friend Support and Adolescent Psychological Distress

Co‐rumination presents a complex construct which can potentially have both positive and negative effects (Stone et al. 2022; Yang et al. 2023). On the positive side, it allows friends to support each other through emotional expression and self‐disclosure, which initially might lead to friends experiencing higher friendship quality and more closeness (Rose 2002; Rose and Rudolph 2006; Rose et al. 2016; Yang et al. 2023). On the negative side, the prolonged focus on problems and negative emotions can lead to ruminative thinking (Bastin et al. 2021; DiGiovanni et al. 2022; Felton et al. 2019) and is linked to an increase in psychological distress both concurrently and over time (Schwartz‐Mette and Smith 2018; Spendelow et al. 2017; Stone et al. 2022; Yang et al. 2023). In addition, research suggests that, when adolescents engage in high level of co‐rumination, daily support seeking (Starr 2015), stress (Bastin et al. 2015; White and Shih 2012) and poor emotional awareness (Miller et al. 2020) are associated with increased depressive symptoms. Furthermore, a recent latent profile study identified distinct subgroups of high co‐ruminators with either higher support and higher depressive symptoms or higher support and fewer depressive symptoms (Tilton‐Weaver and Rose 2023). Building on these findings, this study aims to examine whether relatively high levels of co‐rumination—that is, relative to our sample mean—might dampen the positive effects of perceived best friend support on adolescent psychological distress, while adjusting for prior distress symptoms.

## 2 Gender

The effect of gender on perceived support and co‐rumination seems to present distinct patterns that warrant additional investigation. While not conclusive, some studies suggest that girls may be more dependent on support from close friends concerning their well‐being (Rose and Rudolph 2006; Rueger et al. 2010). Additionally, it is reported that girls are more likely than boys to openly share their thoughts, feelings, and problems with peers (Landoll et al. 2011; Rose and Rudolph 2006). This greater propensity for disclosure extends to co‐rumination. For example, a meta‐analysis shows that girls tend to co‐ruminate more than boys (Spendelow et al. 2017). Importantly, the adverse effects of co‐rumination on psychological outcomes such as anxiety and depression appear to be more pronounced for girls (Rose et al. 2007). These studies highlight the need for gender‐specific testing of the associations between perceived best friend support, co‐rumination, and their impacts on adolescent psychological distress.

Thus, while previous research has focused on apparent negative peer‐processes, such as rejection or peer victimization, as predictors of distress (Ladd and Troop‐Gordon 2003; Rijlaarsdam et al. 2021), whether perceived supportive relationships might contribute to psychological distress when adolescents engage in co‐rumination is not yet clear. Studying the effect of co‐rumination as a moderator on the association between perceived best friend support and distress is important because of its practical implications for the development of interventions focused on perceived best friend support to alleviate adolescents' psychological distress. For example, it is suggested that due to the social benefits associated with co‐rumination, it may be challenging for adolescents to disengage from it without guided support (Rose 2021; Rose et al. 2016; Stone et al. 2022). Furthermore, (public) mental health campaigns such as the Dutch national prevention campaign “Hey it is OK” [‘Hey het is ok’] (Rijksoverheid 2022)‐ or the “Depression: let's talk” campaign (World Health Organization 2017) promote the idea of adolescents seeking advice from friends. Consequently, when co‐rumination indeed diminishes (or even reverses) the positive effect of perceived best friend support, a more nuanced perspective—taking into account potential gender differences, for professionals working in education and youth services as to how adolescents can support each other in a healthy way might be necessary to ensure that such interventions do not inadvertently harm those they intend to help.

## 3 The Present Study

The aim of the present study was to test the potential moderating effect of co‐rumination in the association of perceived best friend support and relative change of symptoms of depression, anxiety, and perceived stress (i.e., psychological distress) from mainstream primary to secondary education among 187 adolescents living in the Netherlands. Given the empirical evidence on support and co‐rumination we hypothesized that higher levels of perceived best‐friend support will predict lower levels psychological distress over time, but only when levels of co‐rumination are relatively low. When co‐rumination levels between best friends are relatively high, we expected that perceived best friend support no longer protects against the development of psychological distress. In addition, we examined a three‐way interaction between social support, co‐rumination, and gender to test if the association between social support and psychological distress varied depending on levels of co‐rumination and differed between boys and girls.

Furthermore, we were specifically interested in the effect of support and co‐rumination within dyadic friendships rather than the effect of having friends in the classroom or being liked by classmates—factors linked to adolescent less distress in past research (Zimmer‐Gembeck and Pronk 2012). Therefore, we accounted for the effects of having a close friend and social preference (being liked *vs.* disliked by peers) in the classroom. In addition, we added gender, age, and educational level as confounding variables because the effect of support and co‐rumination on adolescent psychological distress may be affected by these variables (Chu et al. 2010; Rueger et al. 2016; Salk et al. 2017).

Lastly, additional sensitivity analyses were conducted. One analysis focused on substituting co‐rumination with either co‐brooding or co‐reflection, which are theorized to represent distinct components of co‐rumination. Co‐brooding reflects the more passive and repetitive component, and co‐reflection encompasses the more active, problem‐analyzing component aimed at enhancing understanding (Bastin et al. 2018). Previous studies have linked co‐brooding with an increase and co‐reflection with a decrease in depressive symptoms (Bastin et al. 2014; Bastin et al. 2018). In addition, one previous study found that support from adolescents' best friend can either amplify or buffer the development of symptoms of depression of the friend pair, depending on the initial depression levels of the dyad (van der Mey‐Baijens et al. 2022), indicating that the effect of co‐rumination on the association between support and psychological distress might be stronger for adolescent friends who already exhibit higher levels of psychological distress. Therefore, we also examined whether the moderating effect of co‐rumination varied according to initial levels of psychological distress.

## 4 Methods

### 4.1 Participants

The present study is part of the longitudinal cohort study “Happy Children, Happy Adolescents?” (HCHA), approved by the Medical Ethics Review Committee of the Amsterdam University Medical Centre/VUmc (protocol no. NL37788.029.11). For a comprehensive description of the original study and sample, please see de Wilde et al. (2016) and Tieskens et al. (2018). When children were in the final‐grade of primary school, a follow‐up study in secondary education was conducted. This follow‐up study has been approved by the Medical Ethics Review Committee of the Amsterdam University Medical Centre/VUmc via an addendum on the original HCHA protocol (addendum no. A2017.394, approval date: 3 December 2017).

Depressive symptoms, anxiety symptoms and perceived stress symptoms were assessed at two time points: during the last‐grade of primary school (T1) and during the first or second grade of secondary school (T2). Co‐rumination, perceived best friend support and control variables were assessed once, at T2. For most adolescents, the T2 data were collected in the first‐grade of secondary school (*n* = 106; 56.7%). However, to maintain statistical power, T2 data were also collected in the second‐grade (*n* = 81; 43.3%). Results from independent samples *t*‐tests indicated no significant differences between the T2 first grade and the T2 second‐grade group in measures of psychological distress and perceived support. However, there was a statistically significant difference in the levels of co‐rumination, with the T2 first‐grade group exhibiting a lower mean (*M*_*1grade*_ = 1.84, SD = 0.92) compared to the T2 second‐grade group (M_*2grade*_ = 2.10, SD = 0.72; *t*(184.9) = −2.20, *p* = 0.03 Cohen's d = −0.31).

The sample used in the present study was collected in two consecutive cohorts between March 2017 and July 2019 and measures were assessed once a year. For the current study, only adolescents who provided active written informed consent and had complete data on the psychological distress variables at T2 are included. This resulted in a final sample of 187 adolescents (52.9% girls) spread across 23 secondary schools located in the middle and eastern parts of the Netherlands. Figure S1 shows a flowchart of retention, attrition and reasons for exclusion outlining the selection process of participants in the current sample. Results from independent samples *t*‐tests revealed that compared to excluded adolescents, included adolescents showed on average higher levels of anxiety symptoms (*M*_*included*_ = 0.76, SD = 0.61; *M*_*excluded*_ = 0.66, SD = 0.60; *t*(1449) = −2.240, *p* = 0.025, Cohen's *d* = −0.18 and depression symptoms(*M*_*included*_ = 0.57, SD = 0.42; *M*_*excluded*_ = 0.49, SD = 0.43; *t*(1449) = −2.172, *p* = 0.03, Cohen's *d* = −0.17). However, no significant differences in perceived stress symptoms levels were found between the included and excluded adolescents (*p* = 0.06). Adolescents were on average 13.3 (SD = 0.72) years old in the first‐grade of secondary school and were predominantly (88.0%) of Dutch ethnic background. Only 2% reported low socioeconomic status (20% missing value). As categorized according to the different levels of the Dutch school system, 29% attended “pre‐vocational secondary education”, 47% attended “senior general secondary education” and 24% attended “preuniversity education”.

### 4.2 Procedure

At T1 (primary school), participants and their classmates completed questionnaires on a tablet computer at school during a regular school day, in their classroom. Participants were seated in exam style to ensure privacy. Trained research assistants provided plenary instructions and assisted individual adolescents when necessary.

At T2 (secondary school), participants completed individual questionnaires at home online via a secure, encrypted link that was send to their personal e‐mail address. Each questionnaire was preceded by a standardized instruction. Note that participating adolescents were familiarized with the data collection procedure in previous years. After the assessment, adolescents received a gift voucher of 25 euros. Parents completed a parent questionnaire in which they reported on, among other things, their current educational status as a proxy for socioeconomic status. Classmates of included participants were also invited to participate in a part of the study, to collect—among other constructs—data on social preference. To this end, a team of trained research assistants visited the classroom and provided instructions and individual help when necessary.

### 4.3 Measures

#### 4.3.1 Co‐Rumination

An abbreviated Dutch version (CRQ‐s) of the original 27‐item Co‐Rumination Questionnaire was used (Bastin et al. 2018; Rose 2002). Items assessed to what extent adolescents generally discuss problems with their friend on a five‐point Likert scale (0 = ‘not at all true,’4 = ‘completely true’). Adolescents' mean score was used in the analysis. An example question is: “My friend and I talk almost every time we see each other about problems one of us has.” Previous research shows that the CRQ‐s has good internal consistency, good test‐retest reliability and validity in an adolescent sample (Hankin et al. 2010). Internal consistency in the current sample was good (Cronbach's α = 0.91).

#### 4.3.2 Perceived Best Friend Support

Perceived best friend support was measured using the subscale “Support” of the Network of Relationships Inventory (NRI) (Furman et al. 2002; Van Aken and Hessels 2012). This subscale consists of eight items that adolescents were asked to answer regarding who they considered their best friend using a five‐point Likert scale (0 = “little or none”, 4 = “the most”). Adolescents' mean score of the eight items was used in the analysis, with higher scores indicating higher levels of perceived support. An example item is: “How much does your best friend really care about you?” The original support subscale of the NRI shows good psychometric properties in adolescent samples, such as high internal consistency and moderately high stability over a 1 year period (Furman and Buhrmester 2009). Internal consistency in the current sample was satisfactory (α = 0.89).

#### 4.3.3 Depression and Anxiety Symptoms

Depression and anxiety symptoms were measured with the Generalized Anxiety Disorder and the Major Depressive Disorder subscales of the Dutch version of the Revised‐Child Anxiety and Depression Scale (RCADS; Chorpita et al. 2000; Kösters et al. 2015). The Generalized Anxiety Disorder subscale consists of six items, such as: “I'm worried about things.” The Major Depressive Disorder subscale consists of ten items, such as: “I feel sad or empty.” Adolescents indicated to what extent they suffered from anxiety and depressive symptoms on a four‐point Likert scale (0 = never; 3 = always). The RCADS shows good psychometric properties for all subscales in a sample of children living in the Netherlands (Buil et al. 2023). Cronbach's alpha in the current study for the Generalized Anxiety Disorder subscale was α = 0.87 and for the Major Depressive Disorder subscale α = 0.82. For both subscales the mean score was used in the current analysis.

#### 4.3.4 Perceived Stress Symptoms

Perceived stress symptoms were measured using the Psychological and Physiological subscales of the “Maastricht University Stress Instrument for Children” (MUSIC) (Kraag et al. 2009; Snoeren and Hoefnagels 2014). Items assessed how often adolescents experienced various stressors or reactions to stress over the last week on a four‐point Likert‐scale ranging from 0 = “never” to 3 = “very often”. The psychological stress subscale consists of nine items, such as: “How often in the last week did you find it hard to calm down?” (α = 0.81 of the current sample). The physical stress subscale consists of ten items, such as: “How often in the last week did you get tired without knowing why?” (α = 0.84 in the current sample). Moderate to good internal consistency and test‐retest reliability were found for both subscales in a Dutch adolescent population (Snoeren and Hoefnagels 2014). Cronbach's alpha for the overall perceived stress scale in the current study was α = 0.88. The mean score was used in the current analysis.

### 4.4 Control Variables

#### 4.4.1 Demographic Characteristics

Demographic characteristics included a binary report of gender (self‐reported girl/boy) and age (self‐reported calendar age in years at T2). Additionally, the educational level of the adolescents was obtained from their secondary schools.

#### 4.4.2 Social Preference

Each participant and their classmates completed peer nominations to measure social preference (who they liked and who they disliked the most) in the first or second year of secondary education. For each of the 187 participants a social preference score was computed in accordance with Coie, Dodge and Coppotelli's standard procedure (1982). Higher scores indicate a higher social preference (Bukowski et al. 2012; Coie et al. 1982).

#### 4.4.3 Reciprocal Dyadic Friendship in the Classroom

Dyadic friendships in secondary education were measured through peer nominations (nominate three (best) friends or less from your class). A friendship was regarded as dyadic if both adolescents mutually nominated each other as friend. Each of the 187 participants was then categorized: 0 = no dyadic friendship with classmates and 1 = at least 1 reciprocated dyadic friendship with classmates.

### 4.5 Statistical Analysis

A two‐step procedure was followed to test the hypotheses. In the first step, a path model was used to examine the main effects of co‐rumination and perceived best friend support on psychological distress at T2, while controlling for baseline distress levels measured at T1. All the dependent variables were simultaneously fitted in one model to control for potential covariance between them. In step 2, the interaction term between co‐rumination and support was added as a predictor of the outcome measures (Figure S2). When significant, regions of significance were identified using the Johnson‐Neyman technique (Lin 2020). Moreover, we explored a three‐way interaction between co‐rumination, support, and gender by including the three‐way interaction term in the model during a subsequent step.

Additionally, several sensitivity analyses were conducted. One replaced co‐rumination with co‐brooding or co‐reflection in the moderation model. Another tested a three‐way interaction between co‐rumination, support, and initial psychological distress. Finally, to account for differences in co‐rumination means between T2 first‐ and second‐grade groups, the variable “T2grade” (yes vs. no) was included in the main effect and moderation models. All models were controlled for effects of age, gender, educational level, social preference, and reciprocal dyadic friendship in the classroom.

Models were estimated in Mplus version 8.0 (Muthén and Muthén 1998–2017), using the robust maximum likelihood estimator (MLR) to adjust standard errors for clustering of participants within schools and handle potential non‐normal distributions of data. Missing values were handled via Full Information Maximum Likelihood (FIML). Model fit was determined via the Chi‐square, the comparative fit index (CFI; values ≥ 0.95 = acceptable fit), the root mean square error of approximation (RMSEA, values ≤ 0.06 = acceptable fit), and the standardized root mean square residual (SRMR, values ≤ 0.08 = acceptable fit) (Marsh et al. 2004). Mplus‐code and output is available in the Open Science Framework; OSF, and can be accessed via the following link: https://osf.io/k3yq6/?view_only=f69ee763f25848a1b6a7df0c4585e138.

## 5 Results

Descriptives are presented in Table 1. Correlations between the individual measures of psychological distress, and between each outcome measure across time‐points, were as expected significant and positive. Furthermore, co‐rumination and perceived best friend support were positively correlated. Notably, no correlation was found between best friend support and psychological distress in secondary school.

**Table 1:** Means, standard deviations and range of adolescents' psychological distress, perceived best friend support and co‐rumination and Pearson correlations among study variables.

Variables	*M*	SD	*Min‐Max*	1	2	3	4	5	6	7	8
**Outcome**											
1. Depressive symptoms T2	0.52	0.38	0–1.8	—							
2. Anxiety symptoms T2	0.53	0.45	0–2.3	0.66**	—						
3. Perceived stress symptoms T2	0.70	0.43	0–2.2	0.83**	0.67**	—					
**Predictor**											
4. Depressive symptoms T1	0.57	0.42	0–2.6	0.48**	0.34**	0.43**	—				
5. Anxiety symptoms T1	0.76	0.61	0–3.0	0.40**	0.48**	0.40**	0.67**	—			
6. Perceived stress symptoms T1	0.86	0.45	0–2.3	0.41**	0.31**	0.47**	0.75**	0.62**	—		
7. Co‐rumination T2	1.95	0.85	0–4.0	0.15*	0.17*	0.18*	−0.01	0.04	0.08	—	
8. Perceived best friend support T2	2.55	0.71	0–4.0	−0.05	−0.03	−0.00	−0.08	−0.00	−0.04	0.48**	—

*Note: N* = 187.

* *p* < 0.05

** *p* < 0.01.

### 5.1 Main Effects of Perceived Best Friend Support and Co‐Rumination on Psychological Distress

As shown in Table 2, higher levels of perceived best friend support were associated with lower generalized anxiety symptoms (*B* = −0.12, *SE* = 0.05, 95% CI [−0.22, −0.03], *p* = 0.007, β = −0.20), depression symptoms (*B* = −0.09, *SE* = 0.03, 95% CI [−0.14, −0.03], *p* = 0.003, β = −0.17), and perceived stress symptoms (*B* = −0.07, *SE* = 0.03, 95% CI [−0.12, −0.02], *p* = 0.005, β = −0.12). In addition, higher levels of co‐rumination were associated with a relative increase in anxiety symptoms (*B* = 0.10, *SE* = 0.03, 95% CI [0.03, 0.16], *p* = 0.005, β = 0.19), depression symptoms (*B* = 0.07, *SE* = 0.03, 95% CI [0.01, 0.14], *p* = 0.03, β = 0.17), and perceived stress symptoms (*B* = 0.09, *SE* = 0.04, 95% CI [0.01, 0.17], *p* = 0.03, β = 0.18).

**Table 2:** Main effects of co‐rumination and perceived best friend support on adolescents' psychological distress controlled for T1 psychological distress.

Variables	Depressive symptoms T2		Anxiety symptoms T2		Perceived stress symptoms T2	
	*B*	*SE*	*B*	*SE*	*B*	*SE*
*Main effects*						
Co‐rumination T2	0.07*	0.03	0.10**	0.03	0.09*	0.04
Perceived best friend support T2	−0.09**	0.03	−0.12**	0.05	−0.07*	0.02
*Covariates*						
Gender T2	−0.19***	0.04	−0.22***	0.06	−0.17**	0.07
Age T2	0.09**	0.03	0.09*	0.04	0.07	0.04
Educational level T2	0.07	0.01	0.01	0.02	0.01	0.02
Social preference T2	−0.04	0.03	0.03	0.04	−0.01	0.03
Reciprocal syadic friendships T2	0.24*	0.12	0.18	0.11	0.38**	0.12
Depressive symptoms T1	0.30***	0.05				
Anxiety symptoms T1			0.25***	0.04		
Perceived stress symptoms T1					0.33***	0.05

* *p* < 0.05

** *p* < 0.01

*** *p* < 0.001.

### 5.2 Interaction Effects of Co‐Rumination on the Association Between Perceived Best Friend Support and Psychological Distress

**Figure 1:**
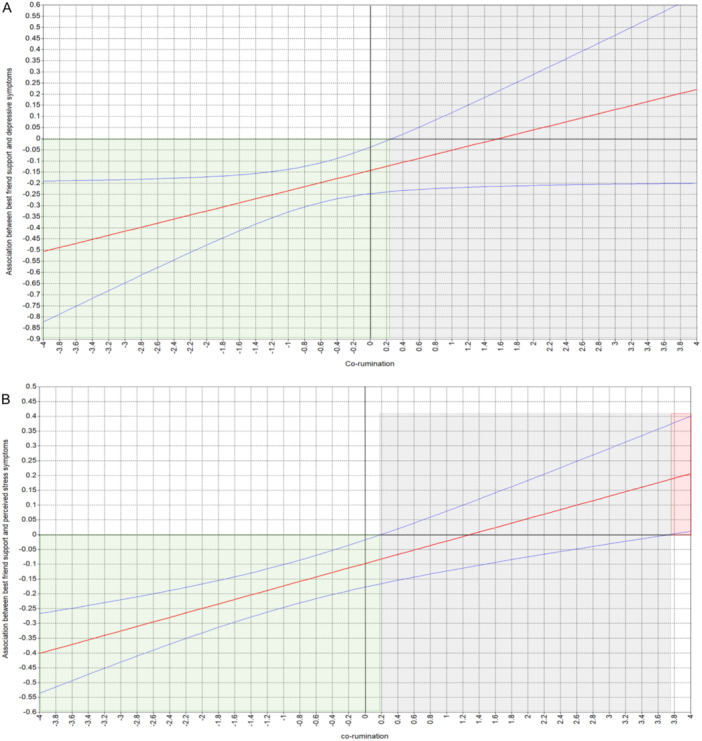
Illustration of the Regions of Significance for the Moderating Effect of Co‐rumination on the Association Between Perceived Best Friend Support and Depressive Symptoms (A) and Perceived Stress Symptoms (B). Note. The straight red plot line represents the adjusted effect of perceived best friend support on either depression or perceived stress symptoms across the full range of co‐rumination values. The blue, curved lines above and below the red plot line represent the 95% confidence intervals for the adjusted effect. The green area indicates where support significantly reduces depression (*z*‐values < 0.26, *p* = 0.048) and perceived stress(*z*‐values < 0.19, *p* = 0.049) symptoms, the gray area denotes nonsignificant effects, and the red area highlights where support significantly exacerbates stress symptoms(*z*‐values > 3.73, *p* = 0.05).

As presented in Table 3, a significant interaction effect between perceived best friend support and co‐rumination was found for depression symptoms (*B* = 0.06, *SE* = 0.03, 95% CI [0.00, 0.11], *p* = 0.05, β = 0.11) and perceived stress symptoms (*B* = 0.06, *SE* = 0.01, 95% CI [0.03, 0.08], *p* = 0.000, β = 0.10), but not for anxiety symptoms (*p* = 0.681). The region of significance (Figure 1) showed that at relatively low levels – that is, relative to our sample mean—of co‐rumination (*z* < 0.19, *p* = 0.049 for perceived stress; *z* < 0.26, *p* = 0.048 for depression), higher perceived best friend support was associated with lower perceived stress and fewer depressive symptoms. However, at co‐rumination above these cutoffs, support was no longer associated with depression symptoms or symptoms of perceived stress. Interestingly, for relatively higher levels of co‐rumination(*z*‐values > 3.73, *p* = 0.05), higher perceived best friend support was associated with higher stress symptoms. Sensitivity analyses examining gender, co‐brooding, co‐reflection, baseline psychological distress, and administration timing of T2 grade revealed no substantial deviations from the main findings, as detailed in Tables S1 and S2.

**Table 3:** Interaction effect of co‐rumination and perceived best friend support on adolescent's psychological distress controlled for T1 psychological distress.

Variables	Depressive symptoms T2		β	Anxiety symptoms T2		β	Perceived stress symptoms T2		β
	*B*	*SE*		*B*	*SE*		*B*	*SE*	
*Main effects*									
Co‐rumination T2	0.07*	0.03	0.16	0.10**	0.04	0.19	0.08*	0.04	0.17
Perceived best friend supportT2	−0.08**	0.03	−0.15	−0.13**	0.05	−0.21	−0.06*	0.03	−0.10
*Interaction effect*									
Support x co‐rumination T2	0.06*	0.03	0.11	−0.01	0.03	−0.02	0.06***	0.01	0.10
*Covariates*									
Gender T2	−0.19***	0.05	−0.27	−0.23***	0.06	−0.26	−0.17**	0.08	−0.21
Age T2	0.10**	0.03	0.19	0.09*	0.04	0.15	0.08	0.04	0.14
Educational level T2	0.01	0.01	0.05	0.01	0.02	0.05	0.01	0.02	0.05
Social preference T2	−0.03	0.03	−0.08	0.02	0.04	0.05	−0.01	0.03	−0.01
Reciprocal dyadic friendships T2	0.26*	0.12	0.19	0.18	0.11	0.11	0.40***	0.11	0.26
Depressive symptoms T1	0.29***	0.04	0.33						
Anxiety symptoms T1				0.24***	0.04	0.34			
Perceived stress symptoms T1							0.32***	0.05	0.35

*Note:*

* *p* < 0.05

** *p* < 0.01

*** *p* < 0.001.

## 6 Discussion

Previous research has found that support from best friends often helps relieve symptoms associated with adolescent psychological distress, including depression, anxiety, and perceived stress. However, co‐rumination, a problem‐focused dyadic emotion regulation strategy (Stone et al. 2022) might diminish this positive effect of friend support. The primary aim of this study was to determine the effect of co‐rumination on the association between perceived best friend support and symptoms of anxiety, depression, and perceived stress in secondary school, while accounting for symptoms exhibited in primary school. When adolescents engaged in relatively low levels of co‐rumination, best friend support was associated with fewer symptoms of depression and perceived stress. At relatively moderate levels of co‐rumination, there was no significant effect of perceived best friend support on symptoms of depression and perceived stress. In addition, at very high levels of co‐rumination (relative to the mean level of co‐rumination in our sample) friend support was associated with higher levels of perceived stress. The analysis accounted for the broader peer‐classroom context and baseline levels of psychological distress. Contrary to what we hypothesized, we found no moderating effect of co‐rumination on the association between perceived best friend support and generalized anxiety symptoms.

Our findings, with the exception of anxiety symptoms, are in part supportive of prior studies on the positive effect of perceived best friend support on adolescent psychological distress (Chu et al. 2010; Gariépy et al. 2016; Schwartz‐Mette et al. 2020). They furthermore support several studies highlighting the moderating role of co‐rumination. Specifically, adolescents who engage in higher levels of co‐rumination report a higher level of depressive symptoms when experiencing interpersonal stress (Bastin et al. 2015) or daily stressful life events (White and Shih 2012). Similarly, higher levels of co‐rumination exacerbate the impact of poor emotional awareness (Miller et al. 2020) and daily problem‐related talk (Starr 2015) on depressive symptoms. These findings emphasize the potential risks of co‐rumination for adolescent psychological distress.

Our findings show that even moderate levels of co‐rumination (relative to the samples mean) undermine the protective effects of friend support on psychological distress. Furthermore, at very high levels of co‐rumination, higher friend support is associated with higher perceived stress symptoms. This supports the idea of co‐rumination as an emotion‐focused coping strategy in which adolescents excessively discuss and dwell on problems and negative emotions that ultimately intensifies rather than alleviates distress (Rose 2002; Rose 2021). Conversely, we found that relatively low levels of co‐rumination (relative to the samples mean), did not impact the association. This aligns with several studies indicating that co‐rumination is not uniformly associated with heightened psychological distress but that its effect depends on context (e.g. high‐quality relationships) (Guassi Moreira et al. 2016), and individual characteristics (Tilton‐Weaver and Rose 2023). For example, and although speculative, adolescents might still frequently disclose to their friends, but without engaging in the repetitive, problem‐focused manner characteristic of co‐rumination. In this case, what might (erroneously be interpreted as) low levels of co‐rumination may instead reflect open, mutual sharing and disclosing that have been shown to foster emotional adjustment and strengthen friendships (Costello et al. 2024; Towner et al. 2022). Alternatively, low levels of co‐rumination might indicate that friends engage in very little disclosure at all. Additional sensitivity analysis that substituted co‐rumination with co‐brooding or co‐reflection (the more repetitive or more active aspects of co‐rumination, respectively) showed that in both cases, relatively moderate levels (relative to the samples mean), diminished the positive effect of perceived friend support on psychological distress. This suggests that engaging in either the repetitive or active components of co‐rumination still disrupts the positive influence of friend support on psychological distress.

Furthermore, we tested via three‐way moderation analysis whether the moderating role of co‐rumination might differ for boys and girls, of which we found no empirical support in our study. This implies that co‐rumination might be a universal risk factor for adolescents, regardless of gender. This is in line with other studies testing sex as a moderator in the association with co‐rumination (DiGiovanni et al. 2022; Hankin et al. 2010). Lastly, several additional sensitivity analyses underscored the robustness of our findings. Models that incorporated initial levels of psychological distress or models adjusting for the timing of T2 data collection, yielded‐overall‐a similar pattern of results as the main model. However, the exacerbating effect of best friend support on perceived stress at very high levels of co‐rumination was not consistently observed across all sensitivity analyses. Furthermore, the interaction effect for depressive symptoms became nonsignificant when adjusting for T2 timing. These findings underscore the robustness of our main results, particularly that at relatively moderate levels of co‐rumination (relative to the samples mean), the positive effect of best friend support on perceived stress is consistently diminished, highlighting the importance of addressing co‐rumination as a potential risk factor for adolescents' psychological distress.

## 7 Limitations

This study has several limitations. First, compared to the original sample of the full research project we encountered a high level of attrition/exclusion of participants for this sub‐study. Furthermore, adolescents included in our sample showed overall low levels of psychological distress, albeit higher than the adolescents not included in our sample. In addition, the sample is composed of adolescents with a predominantly high socioeconomic status (SES). Therefore, our findings may not generalize to other populations, such as adolescents with clinical or subclinical levels of depression and generalized anxiety or samples with a more diverse SES composition. Notably, a recent study suggests that particularly adolescents with lower levels of depressive symptoms who also experience difficulties in shifting their attention away from emotional stimuli, may be particularly prone to co‐ruminate (Rnic et al. 2023). Furthermore, in our study the mean level of co‐rumination was 1.95 (SD = 0.85), which is lower than typically observed in prior studies that used the same questionnaire where means generally exceeded 2.2 [Miller et al. (2020): *M* = 2.22, SD = 0.81; Dirghangi et al. (2015) *M* = 2.82, SD = 1.07; Bastin et al. (2021) *M* = 3.18, SD = 0.74; Schwartz‐Mette and Smith (2018) *M* = 2.9, SD = 0.84]. This might suggest that effects may become apparent at even lower levels of co‐rumination in other studies. Additionally, categorizing gender into only two options failed to acknowledge gender diversity.

Second, while this study measured psychological distress at in primary (T1) and secondary (T2) school, we only had data on co‐rumination and social support in secondary school. As a result, this study cannot provide insights about the development or changes of co‐rumination and support over time, as well as their effect on psychological distress. Furthermore, the measurements in secondary school took place at two different time points, with mean levels of co‐rumination being lower when it was assessed during the first‐grade than during the second‐grade of secondary school (yet, overall co‐rumination means remained low). When controlling our models for the timing of T2, the interaction effect on depressive symptoms was no longer statistically significant (shifting from *p* = 0.047 to *p* = 0.053). Thus, the timing of the assessments appears to have slightly impacted our results. Particularly the effect on depression symptoms does not seem to be robust and should be interpreted with caution.

Third, our findings might be subject to information bias as perceived best friend support, co‐rumination and psychological distress were all based on self‐reports. Although the subjective experience of support and psychological distress arguably are the best way to examine these intra‐personal processes, it can be argued that co‐rumination within peer conversations is ideally assessed using observational data in addition to self‐report (Rose et al. 2014; Rose et al. 2016).

Lastly, there is ambiguity in the friendships we assessed. That is, adolescents were not specifically instructed to report on the same friend for both the co‐rumination and perceived best friend support measures. While the perceived best friend support measure explicitly asked adolescents to focus on their “best friend,” the co‐rumination measure assessed interactions with friends in general. Thus, while it cannot be conclusively stated that the dynamics within a single specific friendship were captured, our study still provides insights on the dynamics of support, co‐rumination, and psychological distress within adolescent friendships. Furthermore, we found no evidence for gender differences in the moderating effect of co‐rumination, but we had no data on whether adolescents had a same‐gender friend in mind or not. We therefore were not able to investigate potential gender differences regarding girl‐girl, boy‐boy, or boy‐girl couples.

## 8 Implications and Future Research

Before drawing any firm conclusions, it is important to replicate the results of this study and establish causal relations first. However, the current research findings ‐ in congruence with others (Spendelow et al. 2017; van der Mey‐Baijens et al. 2022)—suggest that universal prevention strategies targeting adolescent mental health that incorporate best friend support, might want to consider co‐rumination. While adolescents tend to prefer informal help and studies demonstrate the potential benefits of best friend support (Ali et al. 2015; Singh et al. 2019; Smit et al. 2022), these benefits may be decreased or even disappear at when adolescents start to co‐ruminate. Adolescents may therefore benefit from support in recognizing when they are engaging in co‐rumination and learning to flexibly adapt their emotion regulation strategies to fit situational demands by building a diverse repertoire of (dyadic)strategies, such as co‐reappraisal and co‐problem‐solving with friends (Do et al. 2023; Haag et al. 2024).

To better understand whether co‐rumination precedes the development of symptoms of psychological distress or whether there are bidirectional effects, we suggest that future prospective longitudinal studies include multiple measures of co‐rumination and symptoms of psychological distress for a prolonged period. Previous longitudinal studies have shown that co‐rumination, friend support and psychological distress are interrelated constructs and indicate the possibility of bidirectional associations between the constructs (Felton et al. 2019; Rose et al. 2007). However, a longitudinal study conducted over a relatively short period of 6 months, with three timepoints, found concurrent associations between co‐rumination and depressive symptoms, but no longitudinal associations were observed (Harrington 2020). Furthermore, this study did not differentiate between same‐gender and cross‐gender friendships. We suggest that future studies explore the potential impact of a friendship dyad's gender composition as several other studies have found different patterns for same‐gender versus cross‐gender friendships, particularly highlighting a tendency for males to engage more in co‐rumination within cross‐gender friendships (Calmes and Roberts 2008; Hruska et al. 2017). This suggests that examining co‐rumination primarily in same‐gender friendships could underestimate the prevalence among boys, especially in cross‐gender friendships. Lastly, future research could explore, possibly using observational data, the qualitative differences between lower levels of co‐rumination and normative self‐disclosure and examine the nuances between the two in terms of perception and their impact on adolescent psychological distress (Rose et al. 2014).

## 9 Conclusions

The positive effect of perceived best friend support on relieving psychological distress diminishes when adolescents engage in moderate levels of co‐rumination—defined relative to the sample mean. Future studies should aim to establish a causal interaction between co‐rumination and perceived best friend support on adolescent psychological distress. Once established, prevention and treatment programs targeting distressed adolescents could benefit from adopting a (more) nuanced approach to incorporate best friend support in their program. Specifically, these programs should emphasize the value of seeking support from friends while equipping adolescents with a diverse repertoire of dyadic emotional regulation strategies. These strategies might include co‐reappraisal and co‐problem‐solving with friends, helping adolescents adapt their emotion regulation to meet situational demands effectively.

## Author Contributions

**Steffie van der Mey‐ Baijens:** conceptualization, methodology, data‐analysis, writing – original draft preparation. **Patricia Vuijk:** conceptualization, funding acquisition, writing – review and editing. **Kim Bul:** conceptualization, funding acquisition, writing – review and editing. **Pol A. C. van Lier:** conceptualization, writing – review and editing. **Marit Sijbrandij:** supervision, writing – review and editing. **Athanasios Maras:** conceptualization, writing – review and editing. **Marieke Buil:** conceptualization, methodology, data‐analysis, supervision, writing – review and editing. All authors read and approved the final manuscript.

## Ethics Statement

The procedures and measures of the original HCHA project were all approved by the Medical Ethics Review Committee of the Amsterdam University Medical Centre/VUmc (protocol no. NL37788.029.11) and via an addendum on the original HCHA protocol (addendum no. A2017.394, approval date: 3 December 2017).

## Consent

Yearly, active written informed consent for participation and publication was obtained from all participants included in the research project.

## Conflicts of Interest

The authors declare no conflicts of interest.

## Supporting information

Supporting information.

## Data Availability

The data and code necessary to reproduce the analyses presented here are publicly accessible via the following link: https://osf.io/k3yq6/?view_only=70086dd7f5d14e19859d5eafd62a0c72. The materials necessary to attempt to replicate the findings presented here are publicly accessible via the last author: Marieke Buil j.m.buil@vu.nl. The analyses presented here were not preregistered.
