# Supplementary material for: Co‐Rumination as a Moderator Between Best‐Friend Support and Adolescent Psychological Distress
Source: J Adolesc. 2025 Feb 16;97(5):1161–72. doi: 10.1002/jad.12483 (PMC12217408; doi:10.1002/jad.12483)
Supplement: Supplementary file 1 — Supporting information. [file JAD-97-1161-s001.docx]

Online supplemented material

**sFigure 1 Flowchart of Retention and Attrition**


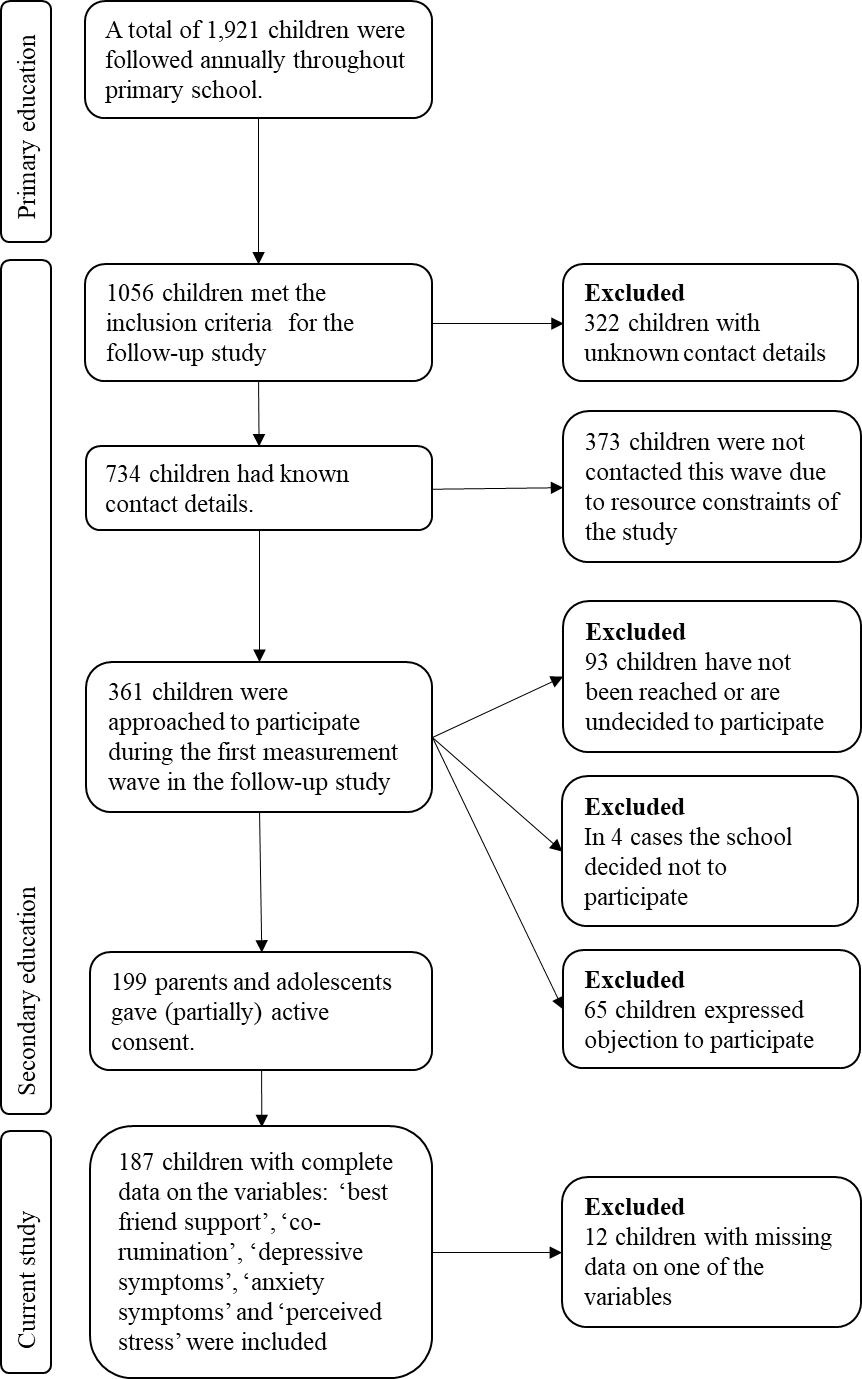


**sFigure 2**

Path Analysis Model of the Moderating Effect of Co-Rumination on the Association Between Perceived Best Friend Support and Psychological Distress


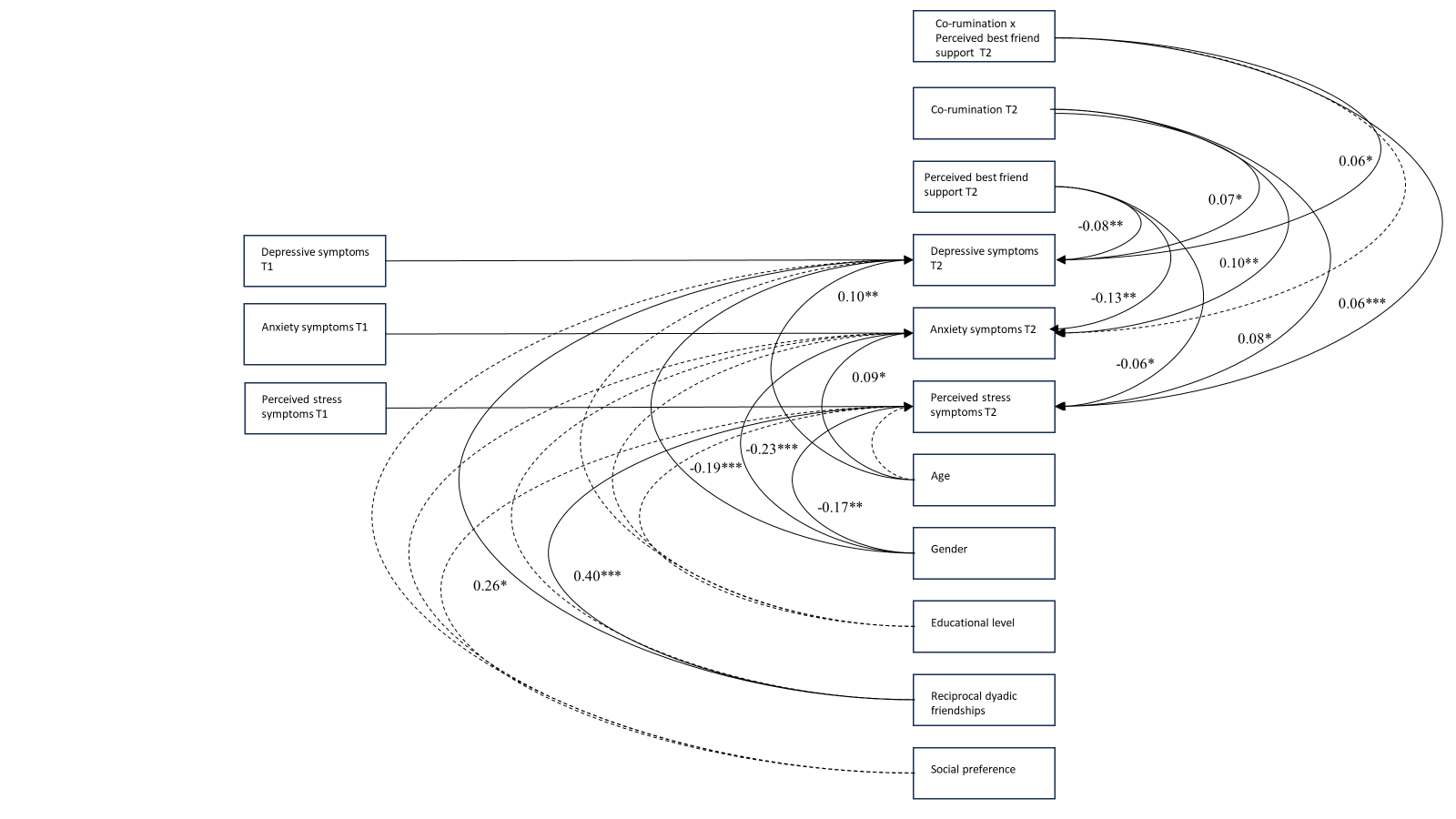


**sTable 1**

*Additional Explorative Three-Way Interactions Regarding Gender and Additional Sensitivity Analysis Regarding Initial Levels of Psychological Distress, Co-brooding and Co-reflection, T2 Data Collection Timing*

|  | | Depressive symptoms T2 | |  | Anxiety symptoms T2 | |  | Perceived stress symptoms T2 | |  |
| --- | --- | --- | --- | --- | --- | --- | --- | --- | --- | --- |
| **Variables** | | *B* | *SE* | β | *B* | *SE* | β | *B* | *SE* | β |
| *Interaction Co-brooding/Co-reflection* | |  |  |  |  |  |  |  |  |  |
| Support x Co-brooding T2 | | 0.06 | 0.03 | 0.22 | -0.01 | 0.03 | -0.04 | 0.05*** | 0.02 | 0.19*** |
| Support x Co-reflection T2 | | 0.09* | 0.05 | 0.11* | -0.02 | 0.04 | -0.02 | 0.08*** | 0.02 | 0.1*** |
| *Threeway interactions* | |  |  |  |  |  |  |  |  |  |
| Support x Co-rumination x Gender T2 | | -0.01 | 0.07 | -0.02 | 0.09 | 0.09 | 0.08 | 0.05 | 0.06 | 0.05 |
| Support x Co-rumination x Initial levels T2 | 0.05 | | 0.08 | 0.04 | -0.08 | 0.10 | -0.06 | 0.09 | 0.06 | 0.06 |

*Note.* The exploratory three-way interaction between support, co-rumination, and gender showed no significant variation between boys or girls. Substituting co-rumination with co-brooding or co-reflection as moderators yielded generally patterns similar to the main analysis. Regarding co-brooding, the interaction for depressive symptoms became non-significant. Including last grade of primary school levels of psychological distress as a moderator revealed no significant influence on the interaction effects.

**sTable 2**

*Interaction Effect of Co-rumination and Perceived best friend support on the Continuation of Adolescent’s Psychological Distress controlled for T2Grade (administration during first vs. second grade).*

|  | | Depressive symptoms T2 | |  | Anxiety symptoms T2 | |  | Perceived stress symptoms T2 | |  |
| --- | --- | --- | --- | --- | --- | --- | --- | --- | --- | --- |
| **Variables** | | *B* | *SE* | β | *B* | *SE* | β | *B* | *SE* | β |
| *Main effects* | |  |  |  |  |  |  |  |  |  |
| Co-rumination T2 | | 0.07* | 0.03 | .16 | 0.10** | 0.03 | .19 | 0.09* | 0.04 | .18 |
| Perceived best friend supportT2 | | -0.08** | 0.03 | -.15 | -0.13** | 0.05 | -.21 | -0.06* | 0.03 | -.11 |
| *Interaction effect* | |  |  |  |  |  |  |  |  |  |
| Support x Co-rumination T2 | | 0.06 | 0.03 | .12 | -0.01 | 0.03 | -.02 | 0.06*** | 0.02 | .10 |
| *Covariates* | |  |  |  |  |  |  |  |  |  |
| T2grade | | 0.05 | 0.07 | 0.07 | -0.02 | 0.09 | -0.02 | 0.13 | 0.07 | 0.15 |
| Gender T2 | -0.19*** | | 0.04 | -.26 | -0.23*** | 0.06 | -.27 | -0.17** | 0.06 | -.20 |
| Age T2 | | 0.07 | 0.05 | .13 | 0.10 | 0.06 | .16 | 0.01 | 0.07 | .02 |
| Educational level T2 | | 0.01 | 0.01 | .04 | 0.01 | 0.01 | .05 | 0.05 | 0.02 | .02 |
| Social Preference T2 | | -0.03 | 0.03 | -.07 | 0.02 | 0.04 | .05 | -0.02 | 0.03 | -.01 |
| Reciprocal Dyadic friendships T2 | | 0.26* | 0.13 | .19 | 0.18 | 0.12 | .11 | 0.41*** | 0.12 | .26 |
| Depressive symptoms T1 | | 0.29*** | 0.04 | .33 |  |  |  |  |  |  |
| Anxiety symptoms T1 | |  |  |  | 0.24*** | 0.04 | .34 |  |  |  |
| Perceived stress symptoms T1 | |  |  |  |  |  |  | 0.32*** | 0.05 | .35 |

*Note.* *p < .05, **p < .01, ***P<.001. Adjusting for the timing of T2 data collection yielded similar interaction effects for perceived stress (B=0.06, SE = 0.02, 95% CI [0.03, 0.09], p < .001, β = 0.11), except for the exacerbating effect at high levels (relatively to our sample), but the interaction for depressive symptoms became marginally non-significant (p=.053).
